# Environmental conditions during winter predict age- and sex-specific differences in reproductive success of a trans-Saharan migratory bird

**DOI:** 10.1038/s41598-017-18497-2

**Published:** 2017-12-22

**Authors:** C. López-Calderón, K. A. Hobson, A. Marzal, J. Balbontín, M. Reviriego, S. Magallanes, L. García-Longoria, F. de Lope, Anders P. Møller

**Affiliations:** 1 Department of Zoology, Faculty of Biology, Green Building, Avenue Reina Mercedes, E-41012 Seville, Spain; 2 Environment and Climate Change Canada, Saskatoon, Saskatchewan, S7N 3H5 Canada; 3 University of Western Ontario, London, Ontario, N6A 5B7 Canada; 4 Department of Anatomy, Cellular Biology and Zoology, University of Extremadura, E-06006 Badajoz, Spain; 5 Ecologie Systématique Evolution, Université Paris-Sud, Orsay, F-91405 France

## Abstract

Previous studies have linked winter habitat with subsequent breeding phenology and physical condition of migratory birds, but few have found delayed effects of winter habitat on subsequent reproductive success. The aim of this study was to test if African winter habitat is related to subsequent reproductive success of house martins (*Delichon urbicum*) breeding at a colony in Spain. We measured stable isotope (δ^2^H, δ^13^C, δ^15^N) values from feathers moulted in West Africa and used confirmatory path analysis to test if isotopic values of winter-grown feathers were related to reproductive success through the mediation of breeding phenology and body condition. We conducted separate analyses for males, females and age classes (yearlings *vs* ≥ 2 years old). Experienced males wintering in habitats of higher rainfall (as inferred from lower feather δ^2^H values) were in better body condition and produced more offspring during the subsequent breeding season. In contrast, we did not find any effect of winter habitat on reproductive success of young males or females. These findings provide evidence consistent with a complex causal link between winter habitat quality and subsequent breeding success of long-distance migratory songbirds.

## Introduction

In an ecological context, carry-over effects occur when the previous history and experience of an individual explains its current performance in a given situation^1^. Many studies of migratory birds have found that environmental conditions experienced during winter have carry-over effects in terms of subsequent body condition and breeding phenology^2–7^. However, few studies have found carry-over effects of environmental conditions from winter areas on final reproductive success^8–12^. For example, older barn swallows (*Hirundo rustica*) arrived earlier to their breeding grounds after winters with favorable environmental conditions and, as a result of increased frequency of second broods, their reproductive success was higher in those years^8^.

Given the difficulty in tracking migratory songbirds with exogenous instruments or markers, analyses of stable isotopes on feathers can provide an alternate or complementary means of studying carry over effects^13,14^. For instance, populations of godwits (*Limosa limosa islandica*) wintering in ^13^C-enriched (i.e., high quality marine) habitats have higher reproductive success than populations wintering in ^13^C-depleted (lower quality terrestrial) habitats^11^.

Environmental conditions experienced in winter may first affect physical condition during winter, timing of departure from wintering sites and timing of arrival to breeding areas^2,4^. Then, arrival time to breeding areas could determine the start of breeding and this in turn directly influence the number of offspring produced^15–17^. In addition, physical condition upon arrival and during the breeding season could be related to breeding performance^18^. Structural Equation Models (hereafter: SEM) and path analysis (a kind of SEM with only measured variables) provide a powerful statistical method to study complex natural systems, since multiple predictor and response variables can be analyzed in a single causal network^19,20^. Specifically, these kinds of analyses can be especially useful in dealing with carry over effects in migratory birds since the above-mentioned variables flow in a time series fashion from wintering to breeding period and vice versa^10^. However, despite the great potential of SEM to detect carry-over effects in migratory birds, it has rarely been used for this purpose. For instance, an indirect carry-over effect of winter habitat quality (inferred from δ^13^C measurements) on reproductive success in American redstart (*Setophaga ruticilla*), was elegantly disentangled by the use of path analysis^10^. In that study, the use of path analysis was essential for discerning that arrival, laying and fledging date, as mediated by the effect of winter habitat quality, determined the number of fledglings finally reared during the subsequent breeding season. A similar result was found for yearling female yellow warblers (*Setophaga petechia*) overwintering in Mexico and breeding in Canada, utilizing general linear models in a path-like analysis^12^. Recently, the complex associations among phenology, winter ecology and breeding performance in the barn swallow have been analyzed by means of partial least squares path modeling (which is related to SEM)^9^.

The house martin (*Delichon urbicum*) is a small colonial hirundine distributed across Europe, Asia and Africa. In the Palearctic, this species breeds across Europe and winters throughout Africa south of 20°N^21,22^, where the single complete annual moult occurs^23,24^. House martins breeding in Western Europe tend to winter in Western Africa and those breeding in Eastern Europe tend to winter in Eastern Africa^25^. Like many aerial insectivores and long-distant migrants, the Palearctic population of house martin has declined during 1990–2000 and the global population is also declining^26–28^. However, causes of these declines and the degree of migratory connectivity in this species are poorly understood^25^. We previously analyzed stable isotope (δ^2^H, δ^13^C, δ^15^N) values from feathers of house martins breeding in southwestern Spain and proposed two winter areas in West Africa with different environmental conditions (savannah and broadleaf forest)^7^. We also found that experienced males winter in savannahs at higher probability than females, whereas young males winter in savannahs at lower probability than females. By contrast, experienced and young females winter in both areas with similar probabilities. Furthermore, we showed that winter area choice was related to clutch initiation date in the subsequent breeding season. However, we did not detect a final carry-over effect on the number of fledglings produced.

The aim of this study was to investigate the relationship between winter habitat of house martins wintering in West Africa and subsequent reproductive success in the temperate breeding area in Southwestern Spain. We used as a proxy of winter habitat the isotopic values of feathers moulted at the wintering grounds, and used confirmatory path analysis to discriminate between some of the likely mediator variables involved in this migratory system. We predicted that environmental conditions in winter areas, as reflected by multiple feather isotopes (δ^2^H, δ^13^C, δ^15^N), indirectly affect the subsequent reproductive success of individuals through effects on subsequent breeding phenology and body condition. Specifically, we hypothesized that open habitats dominated by C4 vegetation with high seasonal rainfall are more suitable for our study species to overwinter than equatorial closed forests dominated by C3 vegetation^7,29^. This is caused by higher rainfall being correlated with higher abundance of flying insects^30–32^, but also because open habitats may provide a better habitat for aerial insectivores to forage. Therefore, we expected that lower feather δ^2^H and higher feather δ^13^C and δ^15^N values would be correlated with earlier breeding phenology, improved physical condition and thus higher reproductive success in the subsequent breeding season. We separately analyzed young (i.e., one-year old birds migrating for the first time) from experienced birds (i.e., two-years or older birds that have previously migrated successfully), and males from females, since we predicted such carry-over effects operate differently according to age and sex^7,12^. Each breeding season, we performed more than one hundred capture sessions from March to July, capturing more than 90% of the colony. Breeding dispersal is negligible for house martins, while recruitment and first reproduction occurs at the age of one year^17,33^. Thus, we could assign the age of individuals accurately, assuming un-ringed adult birds being yearlings at first capture originating from outside the study area, and assuming that disappearance of ringed breeders from the colony indicated mortality rather than dispersal.

## Results

**Figure 1:**
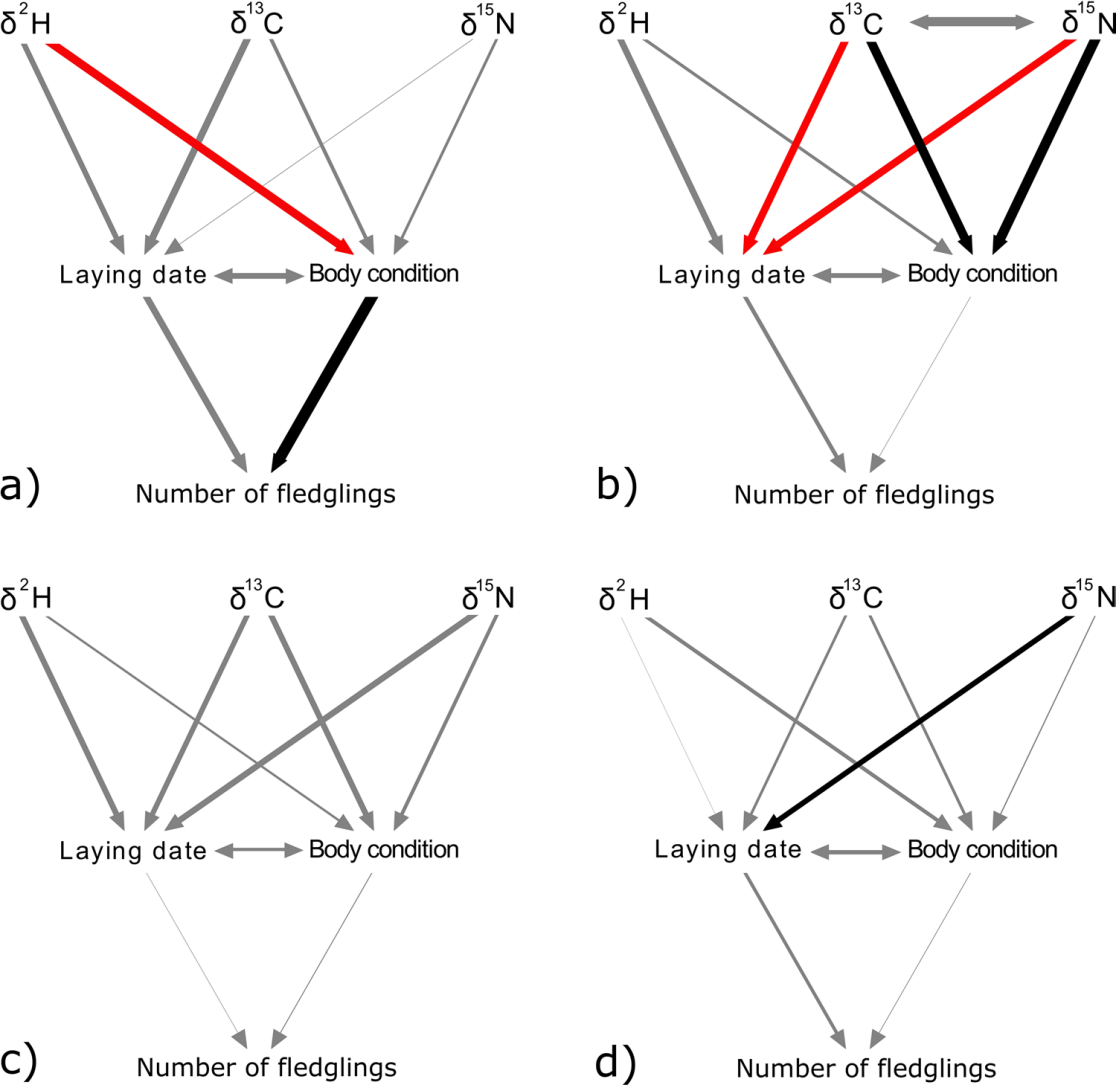
Path diagrams of the models developed for experienced male house martins (**a**), young males (**b**), experienced females (**c**) and young females (**d**). One-headed arrows represent a causal effect of one variable on another (i.e. a path). Double-headed arrows link variables with correlated errors. The width of the arrows reflects the magnitude of standardized path coefficients. Black arrows indicate significant positive effects, red arrows significant negative effects and grey arrows non-significant effects.

Winter habitat features carried over to affect reproductive traits more strongly in males than in females. Breeding performance was affected by different winter habitat features depending on age class. For experienced males, confirmatory path analysis showed that rainfall amount as inferred by feather δ^2^H had a significant direct effect on body condition [estimate (SE) = − 0.33 (0.15), *p* = 0.04], and also that body condition had a significant direct effect on the number of fledglings produced during the breeding season [estimate (SE) = 0.45 (0.16), *p* = 0.01]. For young males, confirmatory path analysis showed that vegetation type as indexed by feather δ^13^C and δ^15^N had significant direct effects on body condition [δ^13^C: estimate (SE) = 0.36 (0.14), *p* = 0.01; δ^15^N: estimate (SE) = 0.40 (0.14), *p* < 0.01] and laying date [δ^13^C: estimate (SE) = −0.29 (0.13), *p* = 0.03; δ^15^N: estimate (SE) = −0.29 (0.13), *p* = 0.04]. For females, we only found that feather δ^15^N had a significant direct effect on the laying date of young females [estimate (SE) = 0.21 (0.09), *p* = 0.03], possibly related to diet (i.e. trophic position) or foraging microhabitat. Therefore, confirmatory path analyses showed strong differences in path coefficients across age and sex categories (Fig. 1, Table 1).

**Table 1:** Summary results from confirmatory path analyses built for each age and sex class. Estimates shown here are standardized path coefficients (i.e. slopes of effects). R^2^ shown here is the conditional R^2^, based on fixed and random effects. Sample size is 38, 28, 52 and 77, respectively, for experienced males, experienced females, young males and young females. Significant effects are highlighted in bold.

Model	Response	Predictor	estimate	SE	P	R^2^
**Experienced males**	No. fledglings	**Scaled body mass index**	**0.455**	**0.160**	**0.010**	0.340
		Laying date	−0.261	0.162	0.136	
	Laying date	δ^2^H	0.217	0.153	0.169	0.418
		δ^13^C	−0.273	0.182	0.160	
		δ^15^N	−0.021	0.163	0.899	
	Scaled body mass index	**δ** ^**2**^ **H**	**−0**.**333**	**0**.**154**	**0**.**041**	0.338
		δ^13^C	0.147	0.180	0.450	
		δ^15^N	0.109	0.163	0.516	
**Young males**	No. fledglings	Scaled body mass index	0.017	0.141	0.908	0.025
		Laying date	−0.161	0.141	0.299	
	Laying date	δ^2^H	0.249	0.141	0.099	0.350
		**δ** ^**13**^ **C**	**−0**.**293**	**0**.**132**	**0**.**034**	
		**δ** ^**15**^ **N**	**−0**.**292**	**0**.**134**	**0**.**037**	
	Scaled body mass index	δ^2^H	−0.122	0.145	0.430	0.340
		**δ** ^**13**^ **C**	**0**.**357**	**0**.**136**	**0**.**013**	
		**δ** ^**15**^ **N**	**0**.**397**	**0**.**139**	**0**.**007**	
**Experienced females**	No. fledglings	Scaled body mass index	−0.034	0.204	0.896	0.002
		Laying date	0.017	0.204	0.939	
	Laying date	δ^2^H	0.229	0.197	0.279	0.205
		δ^13^C	−0.207	0.206	0.379	
		δ^15^N	0.242	0.198	0.244	
	Scaled body mass index	δ^2^H	−0.088	0.194	0.664	0.285
		δ^13^C	−0.229	0.209	0.316	
		δ^15^N	−0.142	0.191	0.472	
**Young females**	Number of fledglings	Scaled body mass index	−0.025	0.118	0.841	0.038
		Laying date	−0.133	0.123	0.369	
	Laying date	δ^2^H	−0.010	0.100	0.923	0.477
		δ^13^C	−0.105	0.094	0.269	
		**δ** ^**15**^ **N**	**0**.**215**	**0**.**094**	**0**.**026**	
	Scaled body mass index	δ^2^H	0.150	0.110	0.179	0.519
		δ^13^C	0.114	0.103	0.273	
		δ^15^N	−0.046	0.103	0.656	

Laying date and body condition were not significantly correlated in any of the confirmatory path analyses [experienced males: estimate = 0.23, *p* = 0.08; young males: estimate = 0.19, *p* = 0.09; experienced females: estimate = −0.13, *p* = 0.75; young females: estimate = −0.17, *p* = 0.93]. Feather δ^13^C and δ^15^N values were not significantly correlated for young males (estimate = −0.37, *p* = 0.99). We used d-separation test to quantify the goodness of fit of our models, which tests the assumption that all variables are conditionally independent^19^. All models provided robust fit to data (experienced males: Fisher’s *C* = 7.7, *df* = 6, *p* = 0.26; young males: Fisher’s *C* = 3.59, *df* = 6, *p* = 0.73; experienced females: Fisher’s *C* = 7.65, *df* = 6, *p* = 0.26; young females: Fisher’s *C* = 6.66, *df* = 6, *p* = 0.35). We did not find any significant association among unconnected variables in any of our models. Thus, we concluded that the hypothesized relationships we examined were consistent with the data. By examining “qq plots”, we determined that every single linear mixed model was well fitted with the only exception of the linear model built for the number of fledglings in experienced females.

**Figure 2:**
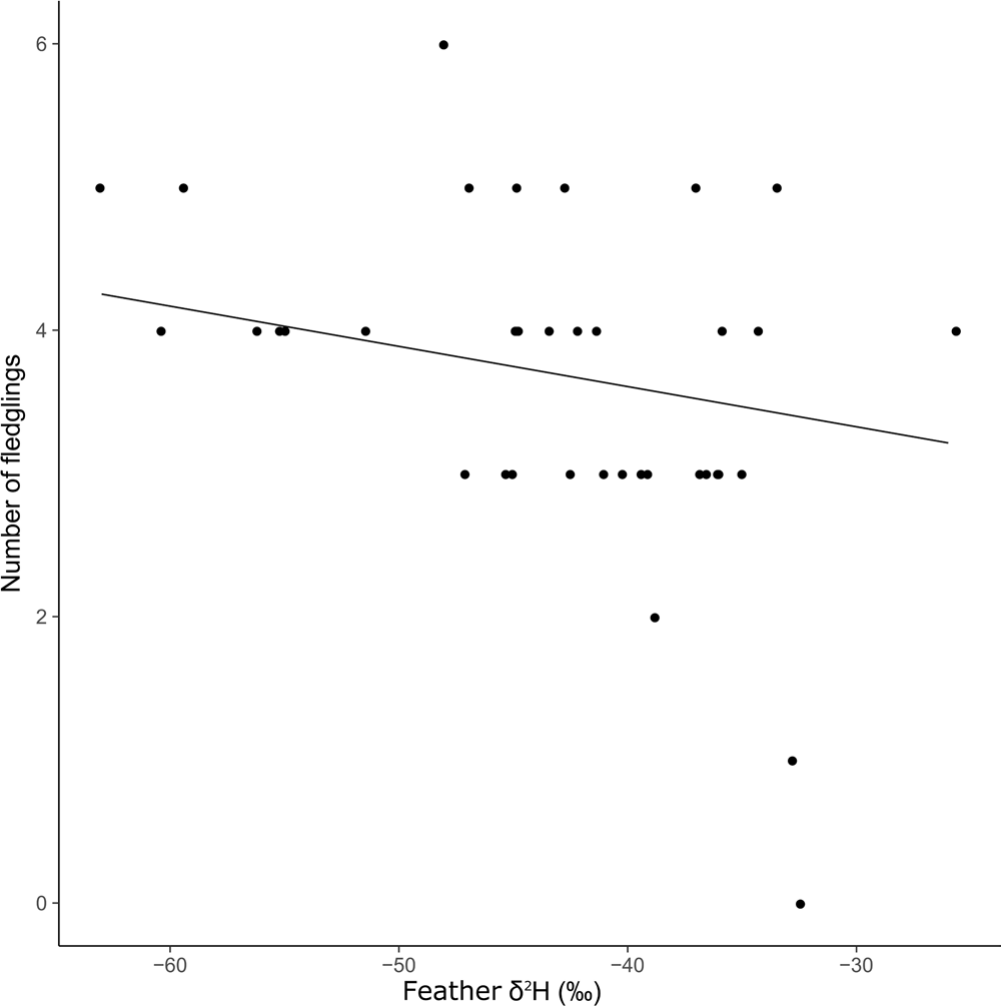
Relationship between feather δ^2^H and reproductive success of experienced male house martins from Badajoz (southwestern Spain). Points represent observed values. The line shows predicted values obtained from the standardized path coefficients of the confirmatory path analysis fitted for experienced males. The increase across feather δ^2^H range of values is predicted to decrease one fledgling reared by experienced males. Predicted values were obtained leaving δ^13^C and δ^15^N constant at their means, and taking into account only fixed effects.

Since for experienced males we found that feather δ^2^H had an indirect effect on the number of fledglings mediated through body condition, we used standardized path coefficients from this model to predict how final reproductive success shifts in response to the change in feather δ^2^H (Fig. 2). We quantified that the increase by one SD of δ^2^H decreases the number of fledglings by 0.2 SD.

## Discussion

We found direct and indirect effects of environmental conditions experienced in the winter areas on different breeding traits in our migratory study population of house martins. Interestingly, we found that environmental conditions experienced during winter in Africa affected reproductive success depending on the age and sex of individuals. We found for experienced males that amount of rainfall, as inferred by feather δ^2^H, directly affected physical condition, and that physical condition directly affected the number of fledglings produced in the first brood. This amounted to a difference of one fledgling reared across the range of feather δ^2^H values. In ecological terms, experienced males wintering in habitats of higher rainfall (i.e. indicated by lower δ^2^H) were in better body condition and raised a larger number of fledglings during the subsequent breeding season than experienced males wintering in drier habitats (i.e. higher δ^2^H). In contrast, vegetation type as indexed by feather δ^13^C and δ^15^N values, affected body condition and breeding phenology of young males. In ecological terms, young males wintering in C4-dominated savannah (i.e. enriched in ^13^C) were in better body condition during the subsequent breeding season and reproduced earlier than young males wintering in broadleaf forest (i.e. depleted in ^13^C). However, the change in these reproductive variables did not translate into a final change in the number of fledglings. This could indicate that breeding phenology and body condition are of lower importance for reproductive success of young compared to experienced males. Actually the confirmatory path analysis built for young males revealed some effect of breeding phenology on the number of fledglings, but not sufficiently strongly to reach statistical significance. We hypothesize that young males may invest less in reproduction than experienced males, yet survival rates of young males are much lower. Characterizing winter habitats based on δ^15^N values are inherently more difficult due to effects of both natural (e.g. climate) and anthropogenic (e.g. fertilizer use, agricultural intensity) factors^34^. Mean predicted δ^15^N values were similar in both isotopic clusters described as winter areas for house martins breeding in Southern Spain^7^. Feather δ^15^N values also affected laying dates of young females, although in the opposite direction as in young males; young females wintering in habitats of higher δ^15^N started to reproduce later than young females wintering in habitats of lower δ^15^N. Because several factors can influence dietary δ^15^N values, including climate, anthropogenic factors and plant nitrogen fixation pathways, it is difficult to predict what may be driving this pattern^34^. Finally, we did not find any effect of winter habitat features on the reproductive performance of experienced females.

Previous studies on Neotropical migratory passerines have found improved reproductive success^10,12^ and improved physical condition associated with lower δ^13^C values expected from more mesic environments such as wet forests or mangroves^2–4,35^. In contrast, previous studies on house martins showed that this species mainly winters in habitats enriched in ^13^C such as open savannah and grassland^29^. We found that experienced males wintering in habitats of presumed higher seasonal rainfall raised more offspring in the subsequent breeding season, and also that young males improved body condition and initiated breeding earlier when wintering in habitats of higher δ^13^C values. Therefore, our results suggest that savannah in West Africa is a more suitable winter area for house martins than broadleaf forest. Previous studies have determined that house martins benefit from higher precipitation on their wintering grounds^36,37^, and this is in agreement with our results based on feather δ^2^H values. We interpret this in terms of higher expected insect production in wet vs dry areas^30–32^.

To our knowledge, only two previous studies have quantified the indirect effect of isotopic signature from the wintering areas on the number of fledglings reared by a small passerine in the subsequent breeding season, and these were both in the New World. The effect of winter habitat on the number of fledglings was stronger for female than for male American redstarts^10^, while only young female yellow warblers experienced this carry-over effect^12^. We also found that environmental conditions from winter areas affected house martins differently according to age and sex. Specifically, a final increase of approximately one fledgling was predicted for experienced males wintering in areas with lowest δ^2^H values relative to areas with the highest values. Interestingly, the final shift in breeding success we found for experienced male house martins was similar to the previous studies on Neotropical migrants^10,12^.

Few studies have identified the specific mechanism underlying carry-over effects^1^. We found previously for house martins breeding in Spain that experienced males winter with a much higher probability in high-quality areas (i.e. savannahs) than young males^7^. However, females winter in high quality areas with similar probabilities regardless of their age category. This finding is consistent with the notion that competition over winter habitats occurs among male house martins, and that it is linked to habitat characteristics important to male reproductive success. Previous studies found that competition at the winter grounds or selective pressure to arrive earlier at the breeding areas are stronger for males than for females^3,4,38–40^, which could explain the differences we found among ages and sexes.

We showed a seasonal carry-over effect in a migratory passerine, where environmental conditions experienced in the tropical wintering areas had an indirect effect on reproductive success. Interestingly, we found this carry-over effect to be operating differently according to age and sex, where only the reproductive success of experienced males is sensitive to isotopic signatures of wintering area foodwebs. Confirmatory path analysis allowed us to identify body condition as a mediator for the observed carry-over effect on reproductive success. The analysis of stable isotopes from feathers has proven very useful for understanding the migratory ecology of this long-distant aerial insectivore, which is difficult or impossible to track by other methods. Further research, ideally involving the ground-truthing of feather isotope values compared to model predictions is recommended.

## Methods

### Field procedures

We studied a single colony of house martins in Badajoz, Southern Spain (38°53’N 7°01’W), during 2005–2013 (excluding 2006). From February to July, we followed reproductive events every second day to determine laying date (i.e., the date the first egg is laid) and brood size (i.e., the number of fledglings in the first brood). Adult house martins were captured and identified with numbered metal rings. Our original sample size was 195 adults and we only took one observation per individual. We categorized our original data in two different age-classes: young birds (i.e., one-year old individuals that have migrated for the first time that year; *n* = 129), and experienced birds (i.e., two-years or older individuals that were at least in their second migration year; *n* = 66). We categorized as young birds, individuals ringed as nestlings/fledglings that were recaptured in the next year (*n* = 41), and also individuals ringed for the first time as adults (*n* = 88). We categorized as experienced birds, individuals ringed as nestlings/fledglings that were recaptured two years or more after their first capture (*n* = 20), and also individuals ringed for the first time as adults that were recaptured in subsequent years (*n* = 46). From each adult we removed the outermost rectrix for isotopic analysis, recorded tarsus length with a digital caliper to the nearest 0.01 mm and body mass with a Pesola spring balance to the nearest 0.5 g. To estimate body condition we used the scaled mass index, which standardizes body mass at a fixed value of a linear body measurement (tarsus) based on the scaling relationship between mass and body length^41^.

### Stable isotope analysis

All feathers were cleaned of surface oils in 2:1 chloroform:methanol solvent rinse and prepared for δ^2^H, δ^13^C and δ^15^N analysis. Deuterium abundance in the non-exchangeable hydrogen of feathers was determined following standard procedures^42^, and using three calibrated keratin hydrogen-isotope reference materials (CBS = −197‰; SPK = −121.6‰; KHS = −54.1‰). Deuterium measurement was performed on H_2_ gas derived from high-temperature (1350 °C) flash pyrolysis of 350 ± 10 µg feather subsamples and keratin standards. Measurement of the three keratin laboratory reference materials, corrected for linear instrumental drift, were both accurate and precise with typical within-run (n = 5) SD values of < 2‰. For feather δ^13^C and δ^15^N analyses, between 0.5 and 1.0 mg of feather material was combusted online using a Eurovector 3000 elemental analyzer (Eurovector, Milan, Italy). The resulting CO_2_ and N_2_ was separated by Gas Chromatograph (GC) and introduced into a Nu Horizon (Nu Instruments, Wrexham, UK - www.nu-ins.com) triple-collector isotope-ratio mass-spectrometer via an open split and compared to CO_2_ or N_2_ reference gas. Using previously calibrated internal laboratory C and N standards [powdered keratin (BWBIII; δ^13^C = −20‰; δ^15^N = 14.4‰) and gelatin (PUGEL; δ^13^C = −13.6‰; δ^15^N = 4.73‰)], within run (n = 5), precisions for *δ* ^15^N and *δ* ^13^C measurements were ~ ± 0.15*‰*. Stable isotope ratios are reported in standard delta (*δ*) notation relative to VSMOW for δ^2^H, VPDB for δ^13^C, and AIR for δ^15^N analyses.

### Environmental conditions in the wintering areas of house martins

House martins have feather δ^2^H values reflecting long-term, amount-weighted, average δ^2^H from precipitation prior to moult according to established calibration equations^43,44^, while δ^13^C and δ^15^N values are associated more directly from inorganic and organic sources to primary production following isotopic discrimination^44,45^. Feather keratin is metabolically inert after synthesis^46^ and so isotopic values in feathers reflect the environmental conditions where they were grown (i.e. the wintering areas). Considerable literature has emphasized that foodweb δ^2^H, δ^13^C and δ^15^N values are strongly influenced by climate^47^. Lower values of δ^2^H in rain are linked to higher amount of precipitation in tropical latitudes^48,49^ (i.e. the so-called “amount effect”). Higher values of δ^13^C are associated with environments dominated by C4 plants as well as C3 plants adapted to hydric stress^50^. Finally, xeric/cultivated habitats tend to be relatively enriched in ^15^N relative to mesic/uncultivated habitats^34^.

**Figure 3:**
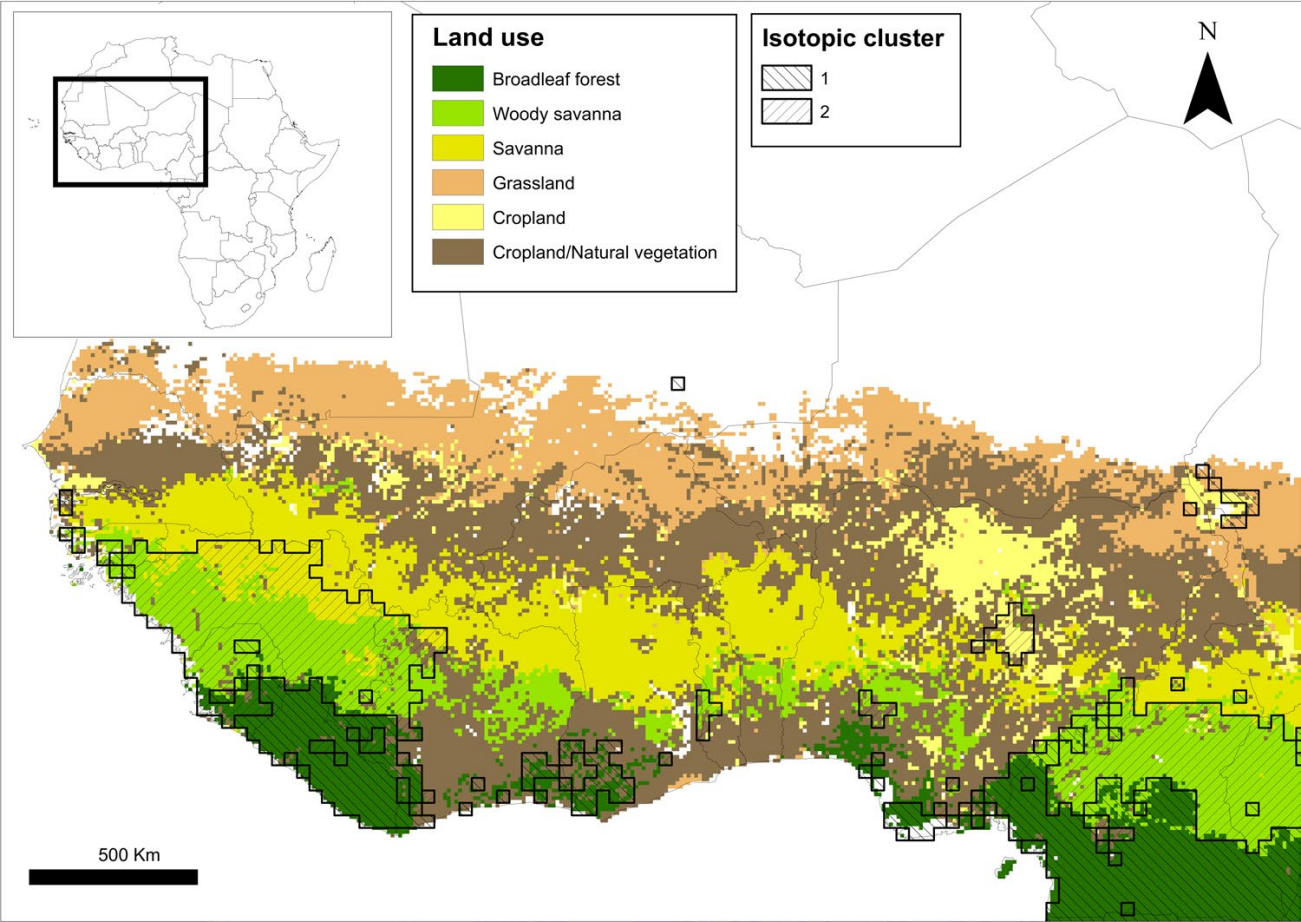
Assigned wintering areas for house martin *Delichon urbicum* breeding at Badajoz (southwestern Spain). African isotopic clusters were generated in ArcGis 10.2.2. (http://support.esri.com/es/Products/Desktop/arcgis-desktop/arcmap/10-2-2), following previously described methods^44^. Land cover classification layer was obtained from freely available images^55^.

The winter areas we previously identified for house martins breeding in southwestern Spain^7^ were defined by multi-isotopic clusters described for Africa^44^. These isotopic clusters were closely associated with different African biomes, broadleaf forest in the so-called “Cluster 1” and savannah in “Cluster 2” (Fig. 3). Values of feather δ^2^H and δ^13^C differed markedly between the two areas of West Africa^7^, reflecting foodwebs in savannahs relatively enriched in ^13^C and depleted in ^2^H. In this wintering area, the savannah receives more rainfall during the rainy season than the broadleaf forest to the south^51^. Thus, we expect the foodweb to be relatively enriched in ^13^C here due to expected C4-dominated grasslands^50^, but depleted in ^2^H due to greater seasonal rainfall^48,49^. Indeed, that pattern was shown previously in the multi-isotopic cluster analysis for Africa^44^.

### Confirmatory path analysis

Structural equation models (SEMs) are probabilistic models that hypothesize a causal network with multiple variables that can appear as both predictor and response variables^20^. SEMs are usually represented as path diagrams in which one-headed arrows represent causal direct relationships from the independent variable to the dependent variable. Path analysis is a type of SEM that only takes into account observed variables; omitting latent variables (i.e. not measured variables^52,53^). We used confirmatory path analysis to examine how feather isotope values reflecting winter habitats could explain reproductive success in the subsequent breeding season through mediation of breeding phenology and body condition. We refer to “confirmatory path analysis” as directed acyclic, or piecewise, SEM based on applications from graph theory^19,20^. In piecewise SEM, the path diagram is translated to a set of linear equations (e.g. linear mixed models), which are then evaluated individually thus allowing a wide range of distributions and sampling designs. Another advantage of this methodology is that it allows fitting models with relatively small sample sizes. In addition, the goodness of fit of the entire causal network can be quantified by a directed separation test (”d-separation test”), which tests the assumption that all variables are conditionally independent (i.e. that there are no missing relationships among unconnected variables^19^).

To control the effect of age and sex on breeding success, we developed different path models for each age and sex category. We split our original data set in four subsets: experienced males (*n* = 38), experienced females (*n* = 28), young males (*n* = 52) and young females (*n* = 77). We included six variables in our models: feather δ^2^H, δ^13^C, δ^15^N, body condition, first-egg laying date and number of fledglings in the first brood. Isotopic values were considered independent (exogenous), while the rest were considered dependent (endogenous) variables. Every observed variable included in our models had the same sample size (i.e. listwise deletion).

The structure of the path analysis was designed based on previous knowledge of the migratory ecology of our study population^7,10^, but also on hypotheses to be tested. Exploratory analyses indicated that δ^13^C and δ^15^N values were negatively correlated for young males. We found in a previous study that breeding phenology (i.e., first egg laying date) depends on wintering area inferred from stable isotope analysis^7^. It is also well known that breeding phenology is related to reproductive success in migratory passerines^6,10^. Different studies have found an effect of wintering habitat (inferred from δ^13^C values) on body condition before departure to the breeding areas^2,4^, and during spring migration^3^. Therefore, we included as hypotheses to be tested causal paths from winter-grown feather δ^2^H, δ^13^C and δ^15^N to body condition during the breeding season. We measured body condition when we first captured the individual. Within our original data set, 140 birds were first captured after they had already started breeding, whereas 55 birds were first captured before they started breeding. For this reason, we assumed correlated errors between laying date and body condition, i.e. a relationship that is bidirectional and assumed to be caused by a shared underlying driver^20^. For instance, arrival time to breeding areas could determine the start of breeding but also the physical condition during the breeding season. The R library *piecewiseSEM* implements a crude approximation of correlated errors by excluding them from the basis set (since there is no presumed direction of causality), and then running a simple test of significance on the bivariate correlation^20^. Finally, body condition may influence the potential foraging of adults and hence the quantity of prey delivery they could afford for their progeny, and this, in turn, may influence the number of fledglings reared. In all cases, we captured adults and measured body condition before we recorded the number of fledglings. Each of our path analyses was made up of three linear mixed models, one for each dependent variable. Isotopic values were the independent variables of the linear mixed models built for body condition and first-egg laying date, while these were in turn the independent variables of the linear mixed model built for number of fledglings. In every linear mixed model we included “year” as the random effect, to statistically control for inter-annual effects. Finally, we examined the “qq plots” to visually test for normality of residuals in every individual linear mixed model. We conducted all our analyses with the library *piecewiseSEM* ^20^ using R version 3.3.1^54^.

### Ethics statement

Methods were evaluated and approved by institutional Commission of Bioethics of University of Extremadura (CBUE 49/2011) and by *Junta de Extremadura* Local Government (CN0008/11/ACA and CN11/0388). All efforts were made to ameliorate suffering of animals and minimize handling time according to Guidelines to the Use of Wild Birds in Research (J. Fair, E. Paul, and J. Jones, Eds. 2010. Washington, D.C.: Ornithological Council). All the experiments comply with the current laws of Spain, where the experiments were performed.

### Data availability

The dataset analysed during the current study is available from the corresponding author on reasonable request.
